# Manual of Diseases of the Eye

**Published:** 1900-12

**Authors:** 


Manual of Diseases of the Eye. For Students and General Practitioners. By Charles H. May, M. D., Chief of Clinic and Instructor in Ophthalmology, Eye Department, College of Physicians and Surgeons, New York. Illustrated. Duodecimo, pp. 419. New York: William Wood & Co. 1900.

The author states in his preface he has endeavored to present a concise, practical and systematic manual of the diseases of the eye. The idea of brevity has been foremost in compiling the book; however, nothing of special interest has been omitted and the average subject is taken up with sufficient detail to make the work valuable to the general practitioner and student.

The description of operations, location of opacities, physiological variations of the normal fundus, relaxation of accommodation, are especially good. Treatment of muscular and refractive troubles are very briefly discussed. The illustrations are good and the whole work shows painstaking effort to make a complete and valuable book of reference.—R. H. S.

